# Multi-stage semi-supervised learning enhances white matter hyperintensity segmentation

**DOI:** 10.3389/fncom.2024.1487877

**Published:** 2024-10-22

**Authors:** Kauê T. N. Duarte, Abhijot S. Sidhu, Murilo C. Barros, David G. Gobbi, Cheryl R. McCreary, Feryal Saad, Richard Camicioli, Eric E. Smith, Mariana P. Bento, Richard Frayne

**Affiliations:** ^1^Departments of Radiology and Clinical Neurosciences, Hotchkiss Brain Institute, University of Calgary, Calgary, AB, Canada; ^2^Calgary Image Processing and Analysis Centre, Foothills Medical Centre, Calgary, AB, Canada; ^3^Department of Biomedical Engineering, Schulich School of Engineering, Hotchkiss Brain Institute, University of Calgary, Calgary, AB, Canada; ^4^Seaman Family MR Research Centre, Foothills Medical Centre, Calgary, AB, Canada; ^5^School of Technology, University of Campinas, Limeira, São Paulo, Brazil; ^6^Department of Medicine (Neurology), University of Alberta, Edmonton, AB, Canada; ^7^Neuroscience and Mental Health Institute, University of Alberta, Edmonton, AB, Canada

**Keywords:** semi-supervised learning, convolutional neural networks (CNN), U-Net, multi-stage learning, white matter hyperintensity (WMH), Alzheimer's disease (AD)

## Abstract

**Introduction:**

White matter hyperintensities (WMHs) are frequently observed on magnetic resonance (MR) images in older adults, commonly appearing as areas of high signal intensity on fluid-attenuated inversion recovery (FLAIR) MR scans. Elevated WMH volumes are associated with a greater risk of dementia and stroke, even after accounting for vascular risk factors. Manual segmentation, while considered the ground truth, is both labor-intensive and time-consuming, limiting the generation of annotated WMH datasets. Un-annotated data are relatively available; however, the requirement of annotated data poses a challenge for developing supervised machine learning models.

**Methods:**

To address this challenge, we implemented a multi-stage semi-supervised learning (M3SL) approach that first uses un-annotated data segmented by traditional processing methods (“bronze” and “silver” quality data) and then uses a smaller number of “gold”-standard annotations for model refinement. The M3SL approach enabled fine-tuning of the model weights with the gold-standard annotations. This approach was integrated into the training of a U-Net model for WMH segmentation. We used data from three scanner vendors (over more than five scanners) and from both cognitively normal (CN) adult and patients cohorts [with mild cognitive impairment and Alzheimer's disease (AD)].

**Results:**

An analysis of WMH segmentation performance across both scanner and clinical stage (CN, MCI, AD) factors was conducted. We compared our results to both conventional and transfer-learning deep learning methods and observed better generalization with M3SL across different datasets. We evaluated several metrics (*F*-measure, *IoU*, and Hausdorff distance) and found significant improvements with our method compared to conventional (*p* < 0.001) and transfer-learning (*p* < 0.001).

**Discussion:**

These findings suggest that automated, non-machine learning, tools have a role in a multi-stage learning framework and can reduce the impact of limited annotated data and, thus, enhance model performance.

## 1 Introduction

White matter hyperintensities (WMHs) are radiological markers commonly observed in brain imaging. They are associated with several neurological conditions including small vessel disease, cerebral ischemia, and neurodegeneration (Duering et al., 2023; Wardlaw et al., 2015). These lesions are commonly observed on routine T2-weighted fluid-attenuated inversion recovery (FLAIR) magnetic resonance (MR) images and are predominantly observed in the periventricular regions, deep white matter, and subcortical areas of the brain. WMH are also evident on T1-weighted (T1w) and T2-weighted (T2w) MR scans as regions of low and high signal, respectively (Wardlaw et al., 2013). The detection and accurate assessment of WMH burden (lesion volume) is important for both clinical management and research studies, providing insights into disease prevalence and, with follow-up imaging, disease progression. A combination of factors, such as the number of WMHs, volume, location and presence of active disease (i.e., new lesions), quantitatively assess WMH progression.

Normally, WMH develops in older adults (>65years) though the lesions are often, initially asymptomatic (Wardlaw et al., 2013). Risk factors such as hypertension, diabetes, smoking, and high cholesterol are associated with development of WMH. These factors, combined with age-related decreases in cerebral blood flow and vessel wall integrity, increase the likelihood of WMH occurrence. Importantly, increased WMH burden is associated with a higher risk of future cognitive decline, stroke, and dementia (Chen et al., 2021; Puzo et al., 2019).

Generating a ground-truth image of WMH is expensive and time-consuming, as it requires experts to manually delineate lesions. Inter-rater reliability can be poor as multiple experts often disagree in their delineation of WMHs, underscoring that identifying and segmenting WMH is a complex and challenging task (Zhu et al., 2022). To overcome this challenge, automated techniques have been proposed that consistently detect and segment those suspected WMH regions seen on FLAIR images. Less commonly T1-w or T2-w images are processed. Machine learning (ML)- and deep learning-based techniques have been identified as promising approaches for segmentation of these lesions. However, the need for large volumes of annotated data in supervised learning approaches negatively impacts the development and generalizability of such tools. Indeed, approaches that can utilize the large volumes of un-annotated FLAIR, T1-w or T2-w available would help to mitigate the need of manual delineation.

Multi-stage semi-supervised learning (MS3L) is an active ML area that uses weakly- (i.e., through automated methods) or un-labeled (i.e., un-annotated) data to build the ML model (Han et al., 2024). We propose using MS3L to improve the training process for WMH segmentation. The term “multi-stage” in MS3L refers to the concept of starting training with lower-quality annotations (termed “bronze” and “silver” standard data) and then progressively refining the model with better-quality annotations (termed “gold” standard data). The term “semi-supervised” describes combining un-annotated data that are segmented automatically, with manually annotated, ground truth, data segmented manually by experts. Our MS3L method initially leverages the large volume of available but un-annotated FLAIR and T1-w scans while still benefiting from the precision of expert annotations later in training.

The goal of this study was (1) to investigate the effectiveness of M3SL in WMH segmentation by training a VGG16-based U-Net variant, and (2) to compare our results against more widely accepted training methods, such as conventional training approaches (our baseline model) and transfer learning (TL)-based approaches. The remainder of this paper is organized as follows: Section 2 provides an overview of related work in the field of WMH segmentation and semi-supervised learning. Section 3 elaborates on the methodology, detailing the (1) proposed multi-stage semi-supervised learning approach, and (2) experimental approach. Section 4 presents our experimental results and performance evaluation, followed by discussions in Section 5. Finally, Section 6 concludes the key findings of the study and provides insight into future research directions.

## 2 Semi-supervised learning for WMH segmentation

Semi-supervised learning (SSL) techniques have been employed to enhance the detection of WMH. One group of studies focuses on combining supervised and unsupervised learning tasks to improve segmentation accuracy. Huang et al. (2023), for example, proposed a semi-supervised level-set loss (LSLoss) approach that leverages FLAIR images and segmentation of brain tissues. They demonstrated significant results in high-resolution images, achieving an average Dice coefficient of 0.83. Similarly, Chen et al. (2019) developed the multi-task attention-based semi-supervised learning (MASSL) method that combines supervised segmentation with unsupervised reconstruction using an attention mechanism. This approach outperformed conventional supervised convolution neural networks and pre-trained models, particularly in applications like brain tumor and WMH segmentation. Yu et al. (2021) further explored MS3L to enhance the fault identification capability of classifiers by combining limited labeled samples with a larger numbers of unlabeled samples. Their study employed data augmentation and metric learning techniques, demonstrating substantial improvements in challenging situations with few labeled samples available.

Another group of studies emphasizes advanced neural network architectures and robust preprocessing techniques to enhance WMH segmentation. Rieu et al. (2021) employed a convolution neural network (CNN)-based model with evolving normalization (EvoNorm) activation layers and MR imaging-based data augmentation techniques. They achieved high accuracy in segmenting various brain regions, including WMH, in FLAIR images. Lee et al. (2023) introduced the AQUA method, a U-Net-based deep learning model with bottleneck attention modules that significantly improved the detection of small lesions and achieved performance comparable to top methods in the MICCAI 2017 WMH Segmentation Challenge (Kuijf et al., 2019).

More recent studies have utilized new deep learning algorithms to advance WMH analysis in MR data, focusing on detection, segmentation, and classification. Zhang et al. (2022) proposed a deep learning algorithm specialized on detecting and segmenting WMH lesions. Their method demonstrated significant improvements in segmentation accuracy across a cohort of 507 patients. Mu et al. (2024) focused on classifying the severity of WMH lesions using deep learning techniques, effectively correlating imaging findings with clinical conditions. Liu et al. (2024) developed a deep learning tool for precise WMH segmentation without requiring manual annotations. Their approach achieved high spatial and volumetric agreement with manual segmentation results from the MICCAI Challenge dataset (Kuijf et al., 2019). Despite these advances, the need for validation on diverse datasets and real clinical scenarios to confirm generalizability remains a common limitation among these studies.

Despite these and other advancements, the need for larger and more diverse datasets to validate generalizability and robustness of proposed methods remains a common limitation. Our work differs from previous studies by specifically exploring the use of MS3L with a unique adaptation of U-Net architecture for WMH segmentation. We aim to refine segmentation quality through iterative training processes that leverage both labeled and unlabeled data to address the challenge of limited available annotated data.

## 3 Materials and methods

### 3.1 Dataset organization and participant information

We employed a combination of three local, annotated datasets (*N* = 260); three publicly available, annotated datasets (*N* = 60); and one publicly available, un-annotated dateset (*N* = 364). The annotated datasets were gathered from five scanner models originating from three different vendors across five centers. FLAIR images were extracted for each individual in the annotated datasets. The un-annotated data were obtained from a multi-center study across many different scanner types from two vendors (Jack et al., 2008). T1-w and FLAIR images were extracted for each individual in the un-annotated dataset. Detailed information regarding the acquisition of each annotated dataset can be found in Duarte K. T. et al. (2023). Details of the un-annotated data can be found in Jack et al. (2008). Tables 1, 2 provide an overview of the participant distribution categorized by sex, age, and clinical stage for each dataset. Table 1 also describes distribution across scanners. Details of how the datasets were organized into training, validation and test sets are provided in Section 3.2.

**Table 1:** Demographics for local and publicly available annotated datasets.

**Dataset**	***N***	**Scanner (see text)**	**Male (%)**	**Age (years)**	**Clinical stage (CN-MCI-AD)**	**nWMH**
**Local dataset**						
CNS_*A*_	74	A	50.0%	31.6 ± 4.4	(74-0-0)	0.03 ± 0.03
CNS_*B*_	20	A	55.0%	43.7 ± 17.3	(20-0-0)	0.06 ± 0.04
FAVR-I	71	A	52.1%	69.7 ± 8.3	(24-29-18)	0.57 ± 0.46
FAVR-II	95	A & B	57.1%	70.7 ± 6.9	(50-26-19)	0.60 ± 0.47
Total	260		53.5%	57.2 ± 7.3	(148-55-37)	
**Public dataset**						
AMS	20	C	N/A^*^	N/A^*^	N/A^*^	0.74 ± 0.69
SIN	20	D	N/A^*^	N/A^*^	N/A^*^	1.37 ± 1.25
Utrecht	20	E	N/A^*^	N/A^*^	N/A^*^	1.44 ± 1.43
Total	60					

Reported are count, percentage, or mean ± standard deviation.

^*^Distribution by clinical stage, sex and age was not reported for the AMS, SIN and Utrecht datasets, though no significant differences by site for age (*p* = 0.45) or sex (*p* = 0.87) were reported (Kuijf et al., 2019).

AD, Alzheimer's disease; CN, cognitively normal; CNS, Calgary Normative Study; FAVR, Functional Assessment of Vascular Reactivity; MCI, mild cognitive impairment; N/A, not available. Normalized WMH (nWMH) is total WMH/total intercranial volume.

**Table 2:** Demographics for publicly available un-annotated dataset.

**Clinical stage**	**ADNI**_*****A*****_			**ADNI**_*****B*****_		
	***N***	**Male (%)**	**Age (years)**	***N***	**Male (%)**	**Age (years)**
CN	69	31.8	76.8 ± 5.5	81	45.6	74.3 ± 7.7
MCI	129	55.0	71.5 ± 5.5	62	56.4	76.3 ± 7.0
AD	21	100.0	77.4 ± 4.4	2	50.0	71.0 ± 2.6
Total	219	58.9	73.6 ± 5.6	145	57.6	75.6 ± 9.0

Reported are count, percentage, or mean ± standard deviation.

AD, Alzheimer's disease; ADNI_*A*_, ADNI data acquired prior to 2017; ADNI_*B*_, ADNI data acquired on or after 2017; CN, cognitively normal; CNS, Calgary Normative Study; FAVR, functional assessment of vascular reactivity; MCI, mild cognitive impairment (aggregate of early mild cognitive impairment; eMCI, late mild cognitive impairment; lMCI, mild cognitive impairment; MCI, and subjective memory concern; SMC; groups reported in ADNI dataset).

#### 3.1.1 Local datasets

The *Calgary Normative Study* (CNS) is an ongoing longitudinal MR investigation concentrating on quantitative imaging techniques in aging (McCreary et al., 2020). The CNS comprises MR images from cognitively normal (CN), community dwelling individuals and were obtained using Scanner A [3 T Discovery MR750, General Electric (GE) Healthcare, Waukesha, WI]. Our sample includes ninety-four individuals selected from this study, divided into younger (CNS_*A*_, *N* = 74, age ≤ 35 years) and older (CNS_*B*_, *N* = 20, age ≥ 40 years) cohorts.

The *Functional Assessment of Vascular Reactivity I* (FAVR-I) study (Peca et al., 2013) provided data from *N* = 71 participants. This single-center observational study explored the connection between cerebral blood flow and cognitive status across clinical stages [CN, mild cognitive impairment (MCI), and Alzheimer's disease (AD)]. FAVR-I data were acquired on Scanner A.

*FAVR-II*, an extension of FAVR-I, is an ongoing, two center study that provided data from *N* = 95 participants (Subotic et al., 2021). FAVR-II involves data acquisition from two scanners: Scanner A (*N* = 65, 68.4%) and Scanner B (*N* = 30, 31.6%; 3 T Prisma; Siemens Healthineers, Erlangen, Germany), situated at a second site.

FLAIR images from all three local datasets underwent segmentation using a semi-supervised approach to generate initial WMH masks. For CNS and FAVR-II, we employed *Cerebra-LesionExtractor* (Gobbi et al., 2012) and FAVR-I used Quantamo (Kosior et al., 2011). These initial WMH masks underwent manual review and editing, as necessary, to produce final ground truth (or “gold” standard) annotated data where each voxel was categorized as either “True” (containing WMH) or “False” (not containing WMH).

#### 3.1.2 Public datasets

We used data from four publicly accessible datasets. Three datasets were part of the 2017 WMH Challenge^1^ (Kuijf et al., 2019) and the fourth was a subset of the Alzheimer's Disease Neuroimaging Initiative (ADNI).^2^ Annotated training data from three distinct sites were obtained from the 2017 WMH Challenge: (1) *Amsterdam* (AMS, *N* = 20)—data acquired using Scanner C (3 T GE Signa HDxt), (2) *Utrecht* (*N* = 20)—data acquired using Scanner D (3 T Philips Achieva; Philips Healthcare, Eindhoven, the Netherlands), and (3) *Singapore* (SIN, *N* = 20)—data acquired using Scanner E (3 T Siemens Trio Tim). Ground truth segmentation masks were provided by the challenge organizers for the AMS, SIN, and Utrecht datasets (Kuijf et al., 2019).

Un-annotated data were also obtained from the *ADNI* database. “ADNI was launched in 2003 as a public-private partnership, led by Principal Investigator Michael Weiner. The primary goal of ADNI has been to test whether serial magnetic resonance (MR) imaging, positron emission tomography (PET), other biological markers, and clinical and neuropsychological assessment can be combined to measure the progression of mild cognitive impairment (MCI) and early Alzheimer's disease (AD).” Selected data from ADNI were used (*N* = 364). These data were extracted using the keyword “MP RAGE” to selected individuals who were imaged with this specific T1-w volumetric imaging technique that was available on scanners from two vendors (Philips and Siemens). ADNI_*A*_ consisted of data acquired using the imaging protocol used over 2010–2016. FLAIR images were acquired in the axial plane using a 2D image acquisition. ADNI_*B*_ included data acquired after 2016 with FLAIR images obtained in the sagittal plane using a 3D acquisition with a phased-array coil. ADNI_*A*_ and ADNI_*B*_ contributed with *N* = 219 and *N* = 145 images, respectively, ranging from CN to AD (Table 2). The total number of individuals available in the full ADNI dataset exceeded the number used in this work. We intentionally selected data acquired on scanners from only two vendors (Phillips, Siemens) to ensure a degree of acquisition protocol and scanner type balance across this study [as Scanners A and C (both General Electric) contributed 230/320 (71.9%) of the annotated data].

#### 3.1.3 Data preparation

To standardize our FLAIR scans, we used the *reorient2std* function from FSL, which applies a rigid-body transformation to align the images with the MNI152 template. We employed the N4 bias-field correction technique (Tustison et al., 2010) to mitigate the impact of intensity variations caused by scanner inhomogeneity. Our acquired 2D FLAIR volumes measured (256 × 256) voxels and had a varying numbers of slices (from 34 to 56 when using 2D FLAIR acquisition, and 256 with 3D FLAIR acquisition). To standardize volume dimensions, we introduced zero-valued (i.e., blank) images to generate consistent dimensions of 256 × 256 × 256. Each image volume was then subdivided into sixty-four equal-sized patches, each measuring 64 × 64 × 64. To tackle class imbalance, we followed the approach outlined by Guerrero et al. (2018). In training, validation, and testing, we exclusively utilized patches containing at least one labeled *True* white matter hyperintensity (WHM) voxel. Most data volumes then underwent normalization by mapping image intensity values to the range (0.0, 1.0). Specifically, we identified the 0th and 98th percentiles of intensity and scaled the entire range accordingly. Notably, the AMS and SIN data exhibited distinct image contrast, suggesting the use of fat suppression during FLAIR image acquisition. For these datasets, we employed min-max normalization using the 0th and 100th percentiles.

Other than converting the T1-w image volumes to standard coordinates, no image preparation was required for the T1-w images used by FreeSurfer, UBO Detector, and Lesion Segmentation Toolbox to calculate bronze standard masks (as described in Section 3.2).

### 3.2 Multi-stage semi-supervised learning (M3SL)

We adopted a M3SL strategy that trained our model with a combination of annotated and un-annotated image data. Our methodology used images acquired in 684 individuals on at least five scanners located at more than five sites, with the un-annotated dataset (Table 2) providing 364/684 (53.2%) of the data. The six manually annotated datasets (Table 1) provided the remaining data. Our strategy consisted of three major training steps:

1. Generation of bronze standard data: We employed standard publicly available image processing toolboxes that can identify lesions in WM on brain MR images. These toolboxes include: (a) *UBO Detector* (Jiang et al., 2018) which identifies small, bright regions in MR images, (b) *Lesion Segmentation Toolbox* (LST) (Schmidt et al., 2012) which employs intensity-based segmentation and machine learning to delineate lesions, and (c) *Freesurfer* (Fischl et al., 2002) which utilize advanced algorithms to segment brain structures for detailed anatomical analysis across all datasets, and can also detect white matter hypointensities in T1-weighted images by applying intensity thresholding within the white matter mask defined by segmentation. These images were registered to the FLAIR for anatomical reference. The current implementation of these techniques employ traditional image segmentation methods and are not significantly influenced by ML algorithms. The masks generated by these tools were termed “bronze” standard because they were not validated by a human expert.
2. Combining with silver standard data: We refined our bronze standard segmentation masks by finding the *consensus* masks using the simultaneous truth and performance level estimation (STAPLE) algorithm (Warfield et al., 2004). STAPLE combined the three bronze masks obtained for each individual and generated a refined WMH mask. These “silver” standard masks represent the consensus of the input masks and provide a better estimate of the true WMH lesions.
3. Refining using gold standard data: In our final stage, we focused exclusively on using annotated data, hence greatly reducing the overall number of images available for training. This stage, referred to as “gold” standard training, fine-tuned our model to capture the most pertinent features specific to WMH segmentation. By training solely on annotated data, we aimed to optimize network performance and refine the ability to discern subtle nuances in the images.

**Figure 1:**
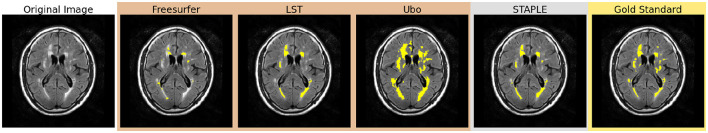
Comparison of bronze, silver, and gold standard segmentations. WMHs are highlighted in yellow in each segmentation.

Through this multi-stage approach, we systematically refined our segmentation model, progressively transitioning from coarser definitions (bronze standard masks) to identifying more specific, nuanced features (gold standard masks). Figure 1 shows a representative case of our training data. By leveraging both un-annotated and annotated datasets in tandem with a purposefully designed training strategy, we aimed to develop a robust and accurate model for WMH segmentation, capable of capturing both overarching image characteristics, as well as intricate details associated with the WMHs within the images.

### 3.3 U-Net model

**Figure 2:**
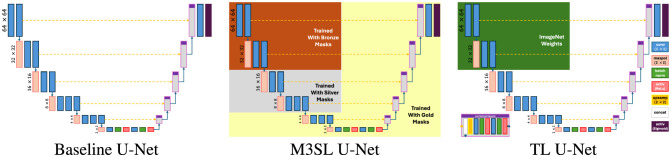
Underlying VGG16-based architecture of the tested U-Net models.

We used a U-Net model implementation that included the VGG16 feature extractor (Simonyan and Zisserman, 2014) in the encoder part, with a mirrored decoding structure and level-wise skip connections between the encoding and decoding layers (see Figure 2). This architecture was chosen based on previous studies that highlighted the effectiveness of VGG16 for accurate WMH segmentation (Duarte K. T. et al., 2023; Duarte et al., 2022). To ensure consistent activation behavior, we employed a sigmoid-shaped activation function and dichotomized the output at a threshold of 0.5. Based on previous findings (Duarte K. T. et al., 2023), we combined the prediction of the axial, sagittal, and coronal results by pooling them (2.5D projection).

In addition to our proposed M3SL model, we varied the training and validation regime to implement two other U-Net variants—*baseline U-Net* (Ronneberger et al., 2015) and *Transfer Learning (TL) U-Net* (Kora et al., 2022)—and evaluated them against M3SL U-Net. The key characteristics and expected strengths of each model are as follows:

1. *Multi-Scale Semi-Supervised Learning (M3SL) U-Net*. This model refines segmentation accuracy through iterative training phases, starting with weakly annotated data and progressively enhancing model performance using a combination of automatically segmented, consensus, and expert-annotated masks (see Section 3.2). The purported strength of M3SL lies in its ability to generalize across diverse imaging protocols, scanners, and clinical stages, making it particularly effective in handling unseen data scenarios.
2. *Baseline U-Net* (Ronneberger et al., 2015). This model features a conventional encoder-decoder structure with skip connections that allow the model to capture fine details in segmentation (Duarte K. T. et al., 2023). The conventional U-Net served as the baseline model for our evaluations. We trained this model only with the gold standard.
3. *Transfer Learning (TL) U-Net* (Kora et al., 2022). This approach leverages a pre-trained model trained on large, unrelated ImageNet-derived dataset (Deng et al., 2009) to improve performance on WMH segmentation tasks. While TL can enhance model performance in data-limited scenarios, its effectiveness may be limited by the differences in data distribution and domain-specific features between the source and target tasks (Zhao et al., 2024). We re-trained this model with the gold standard only.

### 3.4 U-Net model training

**Figure 3:**
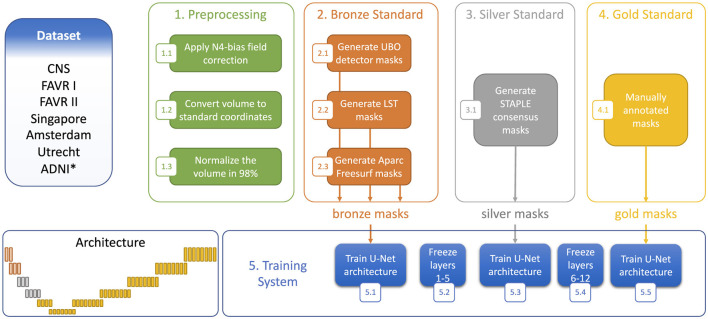
Step-wise training strategy using the three segmentation standards. *Denotes dataset only used to generate the bronze and silver standards.

Our model training utilized a three-stage process involving the sequential use of bronze, silver, and gold standard masks. Figure 3 illustrates the training process, along with the architectural view of the layers corresponding to each segmentation standard. Different data splitting strategies were employed for the bronze, silver and gold standard training. For the bronze standard, the dataset was divided into training and validation sets with an 80%:20% split, omitting a test set as testing was not required at this stage. After freezing the first five layers (Figure 3), the model was retrained using silver standard, consensus-based data, again applying an 80%:20% training:validation division, with the split being stratified to ensure equal representation of MR protocols across the sets. For gold standard training, the first 10 layers were frozen (Figure 3) and a 70%:10%:20% training:validating:test split was used. Finally, to reconstruct the predicted images and thoroughly test the results, the test sets from the five folds of cross-validation were concatenated, ensuring consistency by returning to the same images used at the outset.

We conducted model training with the following parameters: (1) maximum number of epochs: 600, and (2) initial learning rate: $l_{0}=5\times 10^{-4}$. We employed a loss function that considers the sum of dice loss and the binary focal loss. Dice loss is a widely used loss function in the medical field. It accounts for the unequal number of True and False WMH mask elements. The Dice loss was calculated as follows:


(1) $$\begin{array}{c} Dice=\frac{\left(1+\beta^{2}\right)\cdot TP}{\left(1+\beta^{2}\right)\cdot FP+\beta^{2}\cdot FN+FP} \end{array}$$

where β represents the balance coefficient. *TP*, *FP*, and *FN* denote true positive, false positive, and false negative voxel counts, respectively.

The binary focal loss (FL) further addresses class imbalance by adjusting the cross-entropy criterion. The FL was measured by:


(2) $$\begin{array}{c} \begin{array}{c} FL=-GT\alpha {\left(1-PT\right)}^{\gamma }\log\left(PT\right)-\left(1-GT\right)\alpha {PT}^{\gamma }\log\left(1-PT\right) \end{array} \end{array}$$

where *GT* refers to the ground truth and *PT* corresponds to the predicted truth. We adjusted the hyperparameters and found good results for α = 0.25 and γ = 2.0.

We performed our study on a computational cluster comprising four nodes, each equipped with two Tesla V100-PCIE-16GB GPUs and a total memory capacity of 754 gigabytes. Each orientation (axial, sagittial, coronal) was trained independently and in parallel. This approach significantly reduced overall training time. Our models were developed using Python 3.6 within a Jupyter Notebook environment and later converted into Python scripts for execution on the cluster. The complete source code and Keras-based models are freely accessible on GitHub: https://github.com/KaueTND/Margarida_WMH_Seg_Toolbox.

### 3.5 Performance metrics and statistical analysis

When evaluating our models, we encountered a challenge due to the unequal number of True and False WMH voxels (true negative fraction *TNF* ≈0.98, see Supplementary Table S1). Traditional accuracy measures were unsuitable because of the large *TNF*. Instead, we turned to four metrics that do not use true negative counts (*TN*) and are thus less impacted by the imbalance in the data:

- *Precision (P)* (also called positive predictive value) is the ratio of true positive counts to the sum of true positive (*TP*) and false positive (*FP*) counts:
(3) $$\begin{array}{c} P=\frac{TP}{TP+FP} \end{array}$$
- *Recall (R)* (also known as sensitivity) is the ratio of *TP* counts to the sum of true positive and false negative (*FN*) counts:
(4) $$\begin{array}{c} R=\frac{TP}{TP+FN} \end{array}$$
- *F-measure (F)* is widely used performance metric in image segmentation that is the harmonic mean of *P* and *R*:
(5) $$\begin{array}{c} F=2\times \frac{P\times R}{P+R}=\frac{2\times TP}{2\times TP+FN+FP} \end{array}$$
- *Intersection-over-union (IoU)* compares the predicted outcome to the ground truth:
(6) $$\begin{array}{c} IoU=\frac{TP}{FP+TP+FN} \end{array}$$

During training, we saved the model with the highest *IoU* metric as our best model.

We also evaluate our models using the Hausdorff distance (*d*_*H*95_), which quantifies the distance between predicted and ground truth WMH boundaries. Given two sets of points, A and B, the *d*_*H*95_ is defined as:


(7) $$\begin{array}{c} d_{H}(x,y)=max\{d_{AB},d_{BA}\}\\ =max\{\underset{a\in A}{max}\{\{\underset{b\in B}{min}\{d(a,b)\}\},\underset{b\in B}{max}\{\underset{a\in A}{min}\{d(a,b)\}\}\} \end{array}$$

where *d*(*a, b*) represents the Euclidean distance between elements *a* ∈ *A* and *b* ∈ *B*. The 95^*th*^ percentile value of the Hausdorff distance distribution served as our performance metric. In summary, higher *F*-measure and *IoU*, along with lower *d*_*H*95_ values, indicated better model performance.

We used five-fold cross-validation at the gold standard stage to assess the variability of our results and summarized the performance metrics using mean and standard deviation. We also used one-way analysis of variance (ANOVA) tests to evaluate if the mean of the three performance metrics (*F*-measure, *IoU*, *d*_*H*95_) were significantly different based on (1) U-Net variant, (2) disease state (for each variant), and (3) MR scanner (for each variant). A total of seven ANOVA tests were performed for each performance measure. Where appropriate, *post-hoc* Holm-Bonferroni corrected two-sample *t*-tests with pooled variance were applied (α = 0.05).

## 4 Results

**Figure 4:**
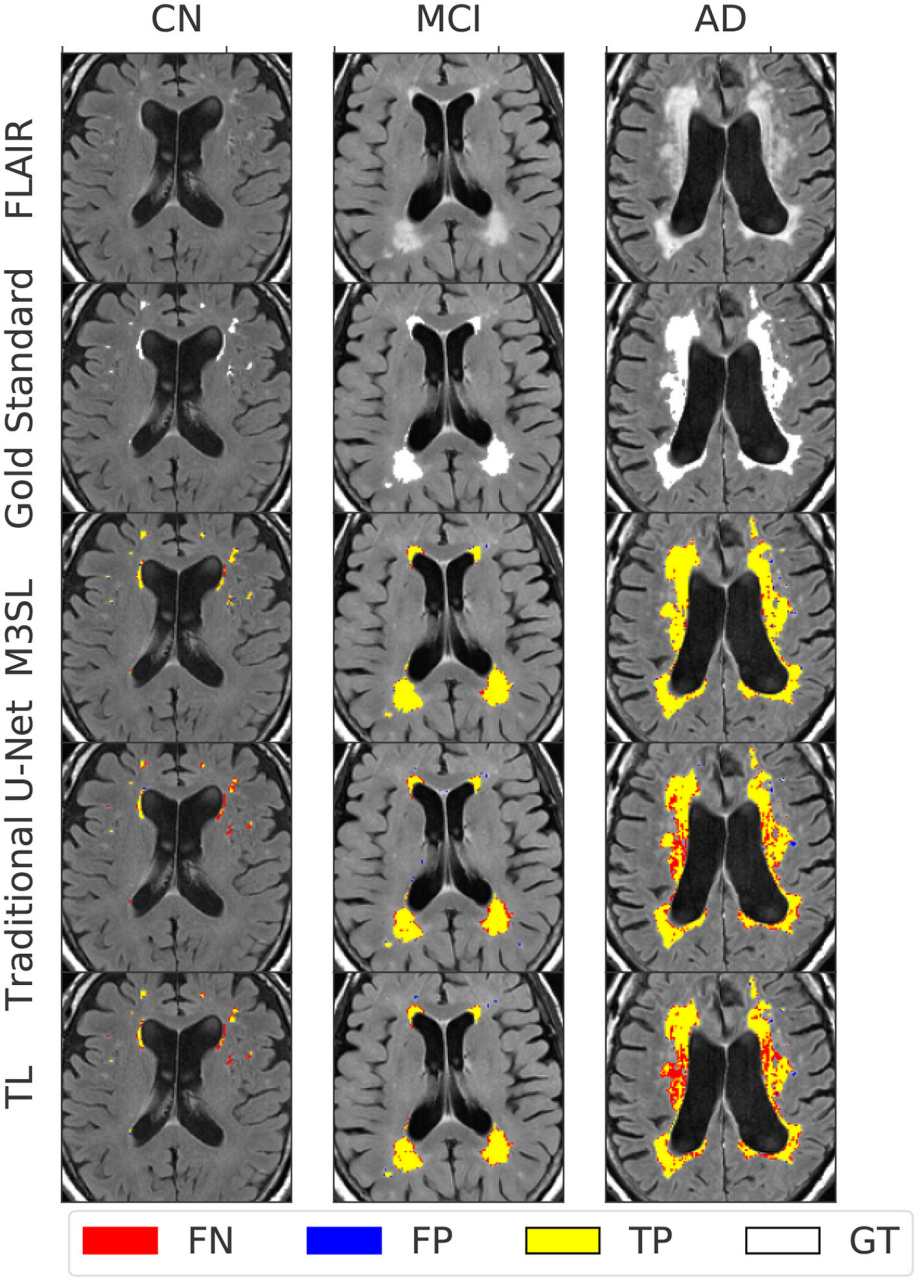
Example segmentation results for each U-Net model at each clinical stage (CN, cognitively normal, 82 year female; MCI, mild cognitive impairment, 84 year female; AD, Alzheimer's disease, 73 year male). Results were obtained using a 2.5D implementation using the VGG16 feature extractor (Duarte K. T. et al., 2023). Total ground truth and M3SL normalized WMH (nWMH) volume for these single images were CN: 0.045% vs. 0.044%, MCI: 0.175% vs. 0.171%, and AD: 0.450% vs. 0.451%, respectively. Supplementary Table S1 summarizes the ground truth-predicted nWMH averaged over all subjects by clinical stage.

Figure 4 presents representative WMH segmentation results and compares the performance of the three evaluated U-Net models (Baseline, TL, and M3SL) across clinical stage (CN, MCI, and AD). Qualitatively each variant performed well in comparison to the ground truth reference (i.e., gold standard mask) segmentation. Most WMH-containing voxels were consistently classified as *TP* across all three models. Across all test data, the resulting normalized WMH (nWMH) values were similar to the gold standard segmentation values (see Supplementary Table S1, Supplementary material). *FN* were reported more frequently than *FP* (Supplementary Table S2), although both counts were less than *TP*. This finding suggests a tendency for all three models to underestimate the ground truth lesions. Closer inspection of Figure 4 confirms the superiority of the M3SL variant which has more *TP* and fewer *FN* and *FP* voxels than the baseline or TL model variants. Despite exhibiting similar trends, the reduction in *FN* and *FP* counts coupled with the increase in *TP* counts was most evident in the M3SL model across the test data (Supplementary Figure S2).

**Figure 5:**
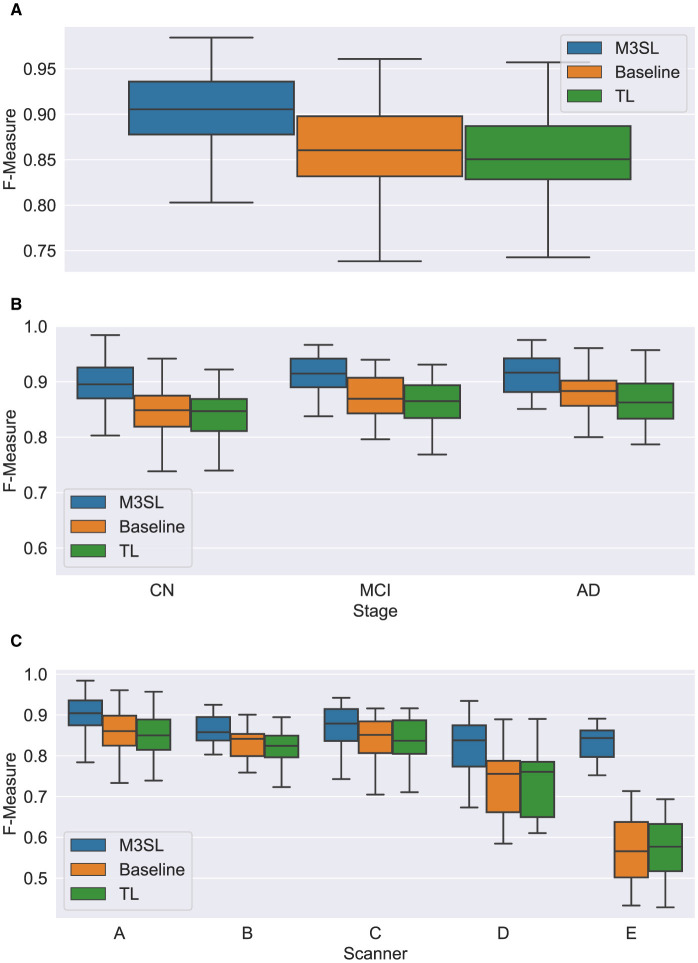
Box plot of *F*-measure score **(A)** across U-Net model variant and by **(B)** clinical stage (CN, cognitively normal; MCI, mild cognitive impairment; AD, Alzheimer's disease), and **(C)** scanner (A–E, see description in text). Outliers have been suppressed to aid visualization.

**Figure 6:**
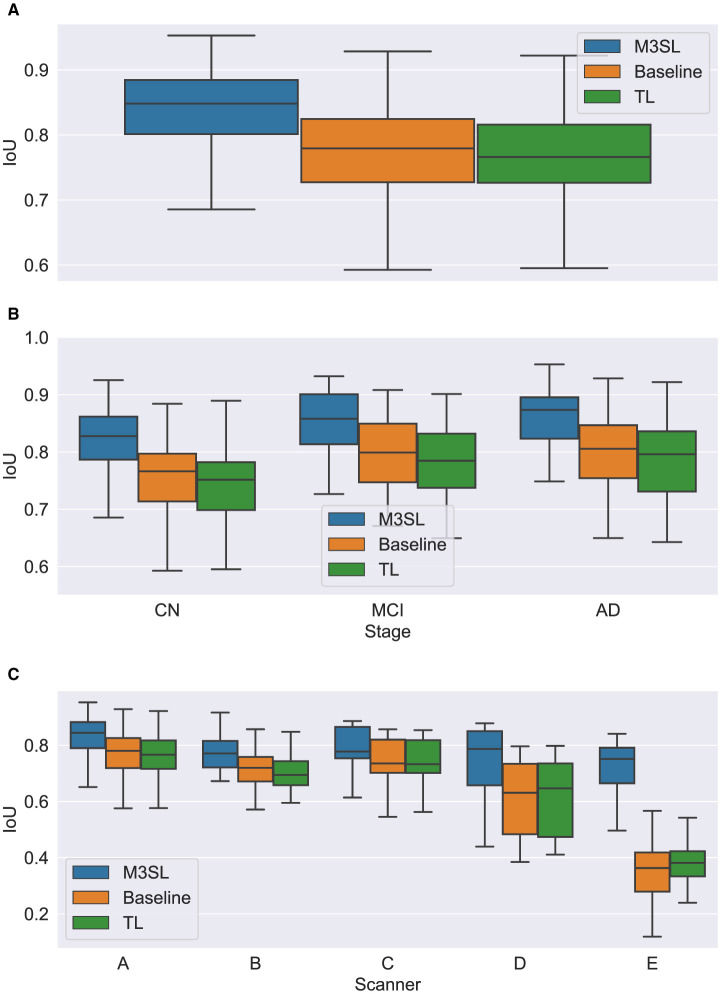
Box plot of *IoU* score **(A)** across U-Net model variant and by **(B)** clinical stage (CN, cognitively normal; MCI, mild cognitive impairment; AD, Alzheimer's disease), and **(C)** scanner (A–E, see description in text). Outliers have been suppressed to aid visualization.

**Figure 7:**
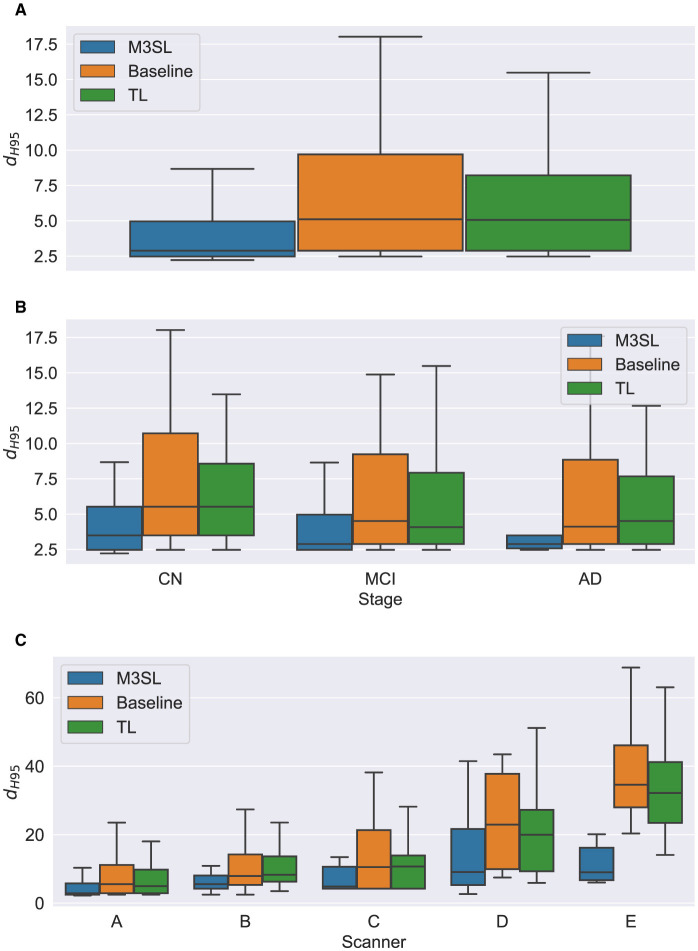
Box plot of Hausdorff 95% percentile distance (*d*_*H*95_) **(A)** across U-Net model variant and by **(B)** clinical stage (CN, cognitively normal; MCI, mild cognitive impairment; AD, Alzheimer's disease), and **(C)** scanner (A–E, see description in text). Outliers have been suppressed to aid visualization.

A summary of the *F*-measure, *IoU*, and *d*_*H*95_ performance metrics for WMH segmentation across the U-Net variants for the 2.5D orientation is presented in Figures 5A, 6A, 7A, respectively. The mean *F*-measures for the three U-Net variants were significantly different [*F*_2,957_ = (36.83), *p* < 0.001, and Supplementary Table S3]. *Post-hoc* *t*-tests demonstrated that the mean *F*-measure for the M3SL model was significantly higher than both the baseline (*p*_*corr*_ < 0.001) and TL (*p*_*corr*_ < 0.001) models. Similarly, the mean *IoU* measures for the U-Net variants were significantly different [*F*_2,957_ = (31.38), *p* < 0.001, Supplementary Table S4]. *Post-hoc* *t*-tests revealed that the mean *IoU* measure for the M3SL model was significantly higher than both the baseline (*p*_*corr*_ < 0.001) and TL (*p*_*corr*_ < 0.001) models. No statistically significant differences in mean *d*_*H*95_ were observed between the models [*F*_2,957_ = (1.548), *p* = 0.213, Supplementary Table S5].

To further explore generalizability, we conducted additional analyses by (1) disease state and (2) MR scanner. Overall, the M3SL model achieved the highest *F*-measure and *IoU*, and the smallest *d*_*H*95_ values (Supplementary Tables S3–S8 for disease state and scanner, respectively).

### 4.1 Model performance by disease state

A summary of the *F*-measure, *IoU*, and *d*_*H*95_ performance metrics for WMH segmentation by disease state for each U-Net variant is presented in Figures 5B, 6B, 7B, respectively. Mean *F*-measure was not significantly different across disease states for both M3SL [*F*_2,131_ = (1.878), *p* = 0.157, Supplementary Table S3] and TL models [*F*_2,131_ = (3.050), *p* = 0.0507]. The mean *F*-measure for the baseline model, however, was significantly different by disease state [*F*_2,131_ = (5.229), *p* < 0.001]. *Post-hoc* *t*-tests revealed that the mean CN *F*-measure for the baseline model was significantly lower than both AD (*p*_*corr*_ = 0.015) and MCI (*p*_*corr*_ = 0.027) values.

Similar findings were observed for *IoU* by clinical stages. Mean *IoU* was not significantly different across disease states for both M3SL [*F*_2,131_ = (2.655), *p* = 0.074, Supplementary Table S4] and TL models [*F*_2,131_ = (2.322), *p* = 0.102]. With the baseline model, the mean *IoU* was significantly different across disease states [*F*_2,131_ = (3.256), *p* = 0.0417]. *Post-hoc* *t*-tests for the baseline model revealed that the mean *IoU* for both AD (*p* = 0.034) and MCI (*p* = 0.038) clinical stages were significantly higher than CN. However, these differences did not survive multiple comparison corrections.

Mean *d*_*H*95_ was not significantly different by disease states for all model variants: M3SL [*F*_2,131_ = (0.805), *p* = 0.449, Supplementary Table S5], TL, [*F*_2,131_ = (0.149), *p* = 0.862], and baseline [*F*_2,131_ = (0.573), *p* = 0.565].

### 4.2 Model performance by scanner

A summary of the *F*-measure, *IoU*, and *d*_*H*95_ performance metrics for WMH segmentation across scanner for each U-Net variant is presented in Figures 5C, 6C, 7C, respectively. The mean *F*-measure was not significantly different across scanner for M3SL [*F*_4,291_ = (1.611), *p* = 0.171, Supplementary Table S6] models. The mean *F*-measure, however, was significantly different across scanner for both TL [*F*_4,291_ = (18.53), *p* < 0.001] and baseline [*F*_4,291_ = (21.77), *p* < 0.001] model variants. *Post-hoc* *t*-tests for the TL variant revealed that the mean *F*-measure for scanner D was significantly smaller than scanner A (*p*_*corr*_ = 0.179), scanner B (*p*_*corr*_ = 0.042), and scanner C (*p*_*corr*_ = 0.024). Scanner D had a larger mean *F*-measure than scanner E (*p*_*corr*_ < 0.001). The mean *F*-measure for scanner E were significantly smaller than scanner A (*p*_*corr*_ < 0.001), scanner B (*p*_*corr*_ < 0.001), and scanner C (*p*_*corr*_ < 0.001). Similarly, *post-hoc* *t*-tests for the baseline variant revealed that the mean *F*-measure for scanner D was significantly smaller than scanner A (*p*_*corr*_ = 0.066), scanner B (*p*_*corr*_ = 0.015), and scanner C (*p*_*corr*_ = 0.013). Mean *F*-measure for scanner D was larger than scanner E (*p*_*corr*_ < 0.001). Mean *F*-measure for scanner E was significantly smaller than scanner A (*p*_*corr*_ < 0.001), scanner B (*p*_*corr*_ < 0.001), and scanner C (*p*_*corr*_ < 0.001).

Mean *IoU* was not significantly different across scanner for M3SL variant [*F*_4,291_ = (1.036), *p* = 0.389, Supplementary Table S7]. Mean *IoU* was significantly different across scanner for both TL [*F*_4,291_ = (18.23), *p* < 0.001] and baseline [*F*_4,291_ = (1.036), *p* = 0.389] model variants. *Post-hoc* *t*-tests for the TL models revealed that the mean *IoU* for scanner E was significantly smaller than scanners A–D (*p*_*corr*_ < 0.001). Similarly, *post-hoc* *t*-tests for the baseline models revealed that the mean *IoU* for scanner E was significantly smaller than all other scanners (*p*_*corr*_ < 0.001).

Mean *d*_*H*95_ was not significantly different across scanner for M3SL model [*F*_4,291_ = (2.239), *p* = 0.064]. However, mean *d*_*H*95_ was significantly different across scanner for both TL [*F*_4,291_ = (9.527), *p* < 0.001, Supplementary Table S8] and baseline [*F*_4,291_ = (10.97), *p* < 0.001] models. *Post-hoc* *t*-tests for the TL model variant revealed that the mean *d*_*H*95_ for scanner E was significantly smaller than scanners A (*p*_*corr*_ = 0.027), scanner B (*p*_*corr*_ = 0.001), and scanner C (*p*_*corr*_ = 0.002). Similarly, *post-hoc* *t*-tests for the baseline models revealed that the mean *d*_*H*95_ for scanner E was significantly lower than scanner A (*p*_*corr*_ < 0.001), scanner B (*p*_*corr*_ < 0.001), scanner C (*p*_*corr*_ < 0.001), and scanner D (*p*_*corr*_ = 0.031).

### 4.3 Result summary

**Figure 8:**
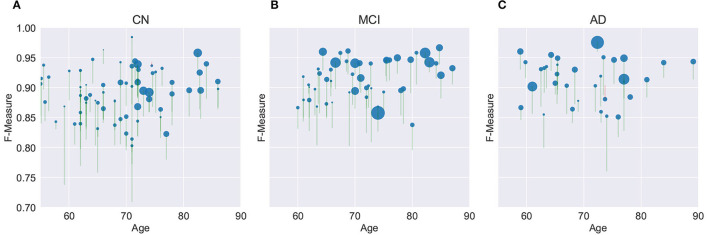
Evaluation of *F*-measure score for the U-Net model across 260 individuals over age grouped by clinical stage: **(A)** CN, cognitively normal (148 individuals), **(B)** MCI, mild cognitive impairment (55 individuals), and **(C)** AD, Alzheimer's disease (37 individuals). Size of the filled circle in the plots reflects rank of the normalized WMH volume (i.e., expressed as a percentage of the intracranial volume). Green vertical lines represent an increase in *F*-measure values in the same individual, from baseline (bottom of the vertical line) to M3SL variant (top of the vertical line). Conversely, red vertical lines indicate a decrease in *F*-measure for the M3SL compared to baseline variant. Only 2/260 (0.007%) of these lines were red. This plot demonstrates better performance for M3SL compared to the baseline model. Data are from the local datasets described in Table 1.

Figure 8 provides a graphical summary of many of the key findings for the M3SL compared to the baseline model for *F*-measure. As would be expected, individuals with larger WMH burdens typically belong to more advanced clinical stages (MCI or AD). Within the CN clinical stage, the expected increase in WMH lesion volumes was observed with advancing age. Larger WMH volumes were associated with higher *F*-measure values, indicating that the M3SL model performed better in cases with a greater disease burden. Figure 8 graphically illustrates that across nearly all individuals (258/260, 99.993%), the M3SL had a higher *F*-measure compared to the baseline variant. Similar findings were seen plotting *IoU* [260/260 (100.0%) improved with M3SL, Supplementary Figure S3] and *d*_*H*95_ [253/260 (99.973%) improved with M3SL, Supplementary Figure S4].

## 5 Discussion

Automating WMH segmentation presents significant challenges due to the variability in lesion volume, irregular shape, and diverse spatial distributions, which may reflect differences in underlying etiology (Duering et al., 2023; Wardlaw et al., 2013). Manual segmentation, though laborious and time-consuming, remains the gold standard but is prone to inter-subject variability. Furthermore, only a limited number of datasets offer annotated/segmented FLAIR, restricting researchers from exploring this topic further (Vanderbecq et al., 2020). Fewer datasets provide WMH-annotated T1-w images. As a result, alternatives that reduce reliance on manual annotation and focus on automated WMH segmentation solutions, even if less accurate, have gained traction (Vanderbecq et al., 2020). While various automated tools are available for WMH segmentation, they often have variable performance when examined across factors such as clinical stage or scanner (Vanderbecq et al., 2020; Wardlaw et al., 2015). Moreover, some tools require T1-w images to define white matter and cerebrospinal fluid boundaries, which may not always be readily available to researchers. Although acquiring FLAIR images alongside T1-w images is a common clinical practice, analyzing them together can potentially increase data management and computational effort due to missing data, registration and other image corrections.

Recently, deep learning techniques for WMH identification and segmentation have emerged as promising alternatives. Deep learning leverages the ability to learn complex patterns from large datasets (Duarte K. T. et al., 2023; Mu et al., 2024). Although transfer learning (TL) offers a practical solution for learning tasks with limited data (Duarte et al., 2019, 2020; Duarte K. T. N. et al., 2023), such as in medical imaging, transferring weights from entirely different domains may not necessarily sufficiently improve model accuracy or generalizability. The inherent differences in data distribution and domain-specific features can limit the effectiveness of pre-trained models (Zhao et al., 2024).

To address these challenges, we proposed M3SL, a robust approach designed to improve segmentation accuracy by iteratively refining the model using a combination of annotated data of varying qualities, including bronze standard data: automatically segmented by non-DL tools (Fischl et al., 2002; Jiang et al., 2018; Schmidt et al., 2012), silver standard data: consensus segmentation via algorithm, such as STAPLE (Warfield et al., 2004), and gold standard data: expert-annotated masks (Kuijf et al., 2019; McCreary et al., 2020; Peca et al., 2013; Subotic et al., 2021). In general the quality of the data would be expected to be in inverse proportion to its availability. Our implementation of M3SL adapts to the unique characteristics of medical imaging data, leading to more accurate and reliable segmentation outcomes in this study. As shown in Supplementary Table S2, a clear increase in True Positive Fraction (*TPF*) highlights the advantages of using the M3SL model. Additionally, the *TPF* of the public dataset showed a marked improvement compared to other U-Net variations, underscoring the importance of using a range of training data to improve generalizability. Other studies have noted efficiency gains by adapting U-Net layers using transfer learning (Kim et al., 2022; Kora et al., 2022; Salehi et al., 2023), but our findings consistently demonstrated that M3SL outperformed conventional methods, including baseline and TL U-Net WMH segmentation models.

One of the key advantages of M3SL is its ability to leverage diverse data sources acquired using different protocols and scanners. The initial layers of a CNN are crucial for extracting general features (Yu et al., 2021). During the bronze standard training phase, our model use data from ADNI. This data accounted for more than 50% of the training data and did not come with gold annotated WMH masks. Exposing the model initially to varied acquisition protocols and multiple scanners likely adjusted the weights and biases of the network, so that this knowledge to be retained by the network. Consequently, the model learned to generalize across a diverse range of images. While the bronze standard data includes lower quality WMH segmentation masks, the subsequent M3SL silver and gold standard stages allow the model to progressively correct these errors (Yu et al., 2021). This adaptability enables the model to learn robust representations of WMHs that are resilient to noise, image artifact and other nonidealities present in the data. The silver standard phase used the STAPLE algorithm (Warfield et al., 2004) to combine the three bronze masks to provide a more accurate, consensus-based mask. This technique has been successfully employed in previous studies (Kats et al., 2019; Warfield et al., 2004) to enhance segmentation quality. Gold standard data was used in the final phase.

The iterative nature of M3SL allows for continuous improvement, refining predictions based on feedback from earlier stages. The M3SL model demonstrated significant improvements in *F*-measure and *IoU* compared to the baseline and TL methods (*p* < 0.001, Figures 5A, 6A, Supplementary Tables S3, S4), providing evidence of superior segmentation performance across the six datasets. When analyzing results by clinical stages (CN, MCI, AD), the M3SL (*p* > 0.05) model showed significant differences in all three performance metrics studied (Figures 5B, 6B, 7B, Supplementary Tables S3–S5). This finding indicates no preference for segmenting FLAIR images at specific clinical stages. In contrast, baseline U-Net models showed higher *F*-Measure and *IoU* in later clinical stages, where the WMH lesions are larger. The M3SL model also showed no significant differences across scanners (*p* > 0.05, Figures 5C, 6C, 7C, Supplementary Tables S6–S8). This finding highlights the advantage of incorporating a large public dataset (ADNI) during the bronze and silver training steps. However, both TL and baseline U-Net models exhibited decreased *F*-measure values with scanner E, as evidenced in Figures 5C, 6C, 7C, with at least a 20% difference in mean *F*-measure value.

Generalization in deep learning models is crucial for ensuring reliable predictions when processing unseen data. Researchers and developers must consider the robustness of WMH predictions across various factors, including clinical stage (*e.g*., CN *vs*. MCI *vs*. AD), WMH size and location, and the acquisition protocol and scanner used to acquire the FLAIR images (Meng et al., 2022). Investigating the WMH burden, particularly in the periventricular region, is important because of the suggested association with WMH burden and increased risk of cognitive decline. The literature on this topic remains somewhat inconclusive on whether these lesions contribute to or result from the onset of dementia. Access to larger datasets will help address these questions provided that appropriate tools exist to accurately segmented images to produce WMH masks.

Some of the main limitations addressed by this study are the poor availability of both annotated public and more advanced disease-containing datasets. While there are abundant FLAIR scans in public repositories (*e.g*., ADNI), the integration of manually annotated ground truth (or “gold” standard) data remains a bottleneck. Additionally, the lack of extensive datasets that account for other pathologies leading to WMHs further constrains the generalizability of the findings. Furthermore, it is important to note that datasets such as ADNI exclude participants with severe cerebrovascular disease (CVD), and while WMH is present, it is not a major contributor to dementia in those cohorts. The CNS cohorts, similarly, are too young to show significant WMH burden. Moreover, not explicitly considering factors such as age and sex can introduce potential biases, which may or may not impact the robustness of the results.

## 6 Summary and conclusions

Although the clear quality of baseline and TL U-Net models, M3SL has shown significant potential in improving segmentation performance by fine-tuning the weights in the initial convolutional layers using broadly weakly-annotated data. This adjustment enhances the model's ability to generalize when encountering diverse image types, including different pulse sequences, acquisition protocols, and preprocessing variations. Our experiments demonstrated that M3SL outperformed baseline U-Net methods (*F*-measure, *p*_*corr*_ < 0.001) and TL-based approaches (*F*-measure, *p*_*corr*_ < 0.001). Similar improvements were observed in the *IoU* metric. When comparing our model with conventional training methods, M3SL exhibited superior generalization to unseen scenarios, such as data from different acquisition protocols and scanners. Additionally, M3SL demonstrated reduced *FP* and *FN* fractions compared to the baseline and TL methods while achieving a significant increase in the *TP* fraction across all clinical stages and datasets.

The performance of the *F*-measure, *IoU*, and *d*_*H*95_ metrics in M3SL was not influenced by clinical stage. However, the baseline U-Net architecture showed a bias toward more severe clinical stages. By grouping and highlighting presumed lesion locations according to clinical stage, our results indicated that M3SL did not differentiate based on clinical stage or lesion volume, as evidenced by the consistent improvement in *F*-measure with disease progression. Differences in acquisition protocol and scanner had no significant impact on the performance of M3SL. This finding addresses a common challenge in deep learning-based WMH segmentation, where models often require access to diverse datasets during the training phase to achieve robust performance.

However, while WMH segmentation provides valuable information, it is not sufficient on its own for comprehensive clinical insights. Understanding the location and temporal progression of WMHs is crucial for a more complete assessment. Our future goal is to utilize longitudinal analysis to explore the potential of WMHs as predictive tools for conditions such as Alzheimer's disease, vascular dementia, and related disorders. To achieve this, future studies should aim to expand dataset diversity by including a variety of temporal information from patients.

## Data Availability

The original contributions presented in the study are included in the article/Supplementary material, further inquiries can be directed to the corresponding author.

## Footnotes

^1^ https://wmh.isi.uu.nl

^2^ https://adni.loni.usc.edu/
