# Supplementary material for: Multi-stage semi-supervised learning enhances white matter hyperintensity segmentation
Source: Front Comput Neurosci. 2024 Oct 22;18:1487877. doi: 10.3389/fncom.2024.1487877 (PMC11534601; doi:10.3389/fncom.2024.1487877)
Supplement: Supplementary file 1 [file Data_Sheet_1.PDF]

Supplementary Material

Multi-Stage Semi-Supervised Learning Enhances White Matter Hyperintensity Segmentation

Kauê TN Duarte [1,2], **Abhijot S Sidhu** [3,4], **Murilo C Barros** [5], **David G Gobbi** [1,2], **Cheryl R McCreary** [1,4], **Feryal Saad** [1], **Richard Camicioli** [6], **Eric E Smith** [1], **Mariana P Bento** [3], **Richard Frayne** [1,2,3,4]

[1]Radiology and Clinical Neurosciences, Hotchkiss Brain Institute, University of Calgary, Calgary, Alberta T2N 1N4, Canada
[2]Calgary Image Processing and Analysis Centre, Foothills Medical Centre, Calgary, Alberta T2N 2T9, Canada
[3]Graduate Program in Biomedical Engineering, Hotchkiss Brain Institute, University of Calgary, Calgary, Alberta T2N 1N4, Canada
[4]Seaman Family MR Research Centre, Foothills Medical Centre, Calgary, Alberta T2N 2T9, Canada
[5]School of Technology, University of Campinas, Limeira, São Paulo, 13484-332, Brazil
[6]Medicine (Neurology), University of Alberta, Edmonton, Alberta, T6G 2R7, Canada

1 SOFTWARE DESIGN

The **Margarida White Matter Hyperintensities (WMH) Segmentation Toolbox** is a comprehensive tool designed to facilitate the segmentation of FLAIR images using variations of the U-Net architecture. The software provides an intuitive interface for researchers and clinicians to load and process medical images, customize segmentation settings, and obtain binary masks for analysis.

User Interface Overview

The main screen of the Margarida WMH Segmentation Toolbox (Figure S1) is divided into two primary areas:

- **Right Panel:** The right area features a 2×2 grid for displaying images. It allows users to navigate through different image orientations (axial, coronal, sagittal) and visualize the segmentation process in real-time. Users can scroll through the slices of the loaded FLAIR volume using this panel.
- **Left Panel:** The left area contains the control menu, which includes several key functions:
  - **Load FLAIR Volume:** This button allows the user to load the FLAIR volume into the software for segmentation.
  - **Load FLAIR Mask (Optional):** Users can load a corresponding manual mask, if available, to compare against the automated segmentation results.
  - **Image Orientation:** This dropdown menu offers different orientations for conducting the segmentation: 2DAxi (axial), 2DCor (coronal), 2DSag (sagittal), and 2.5D (combined orientation).
  - **Model Variant:** This field allows users to select from various U-Net variants for segmentation. This field will be updated with new techniques as they are developed and evaluated, users are advised to check the latest options on the official GitHub repository.

- **Base Model Architecture:** Users can choose from four different commonly used architectures: VGG16, VGG19, ResNet (152 layers), and EfficientNet B0.
- **Threshold Slider:** This slider controls the probability threshold for dichotomizing the segmentation mask, with values ranging from 0 to 1.
- **Study Type:** Currently, the toolbox supports a cross-sectional model, with plans to include a longitudinal approach in future updates.
- **Generate Mask:** This button runs the selected U-Net model to generate the segmentation mask.
- **Generate PDF:** Exports a detailed report of the segmentation results as a PDF (Figure S2a).
- **Run Multiple Patients:** This feature allows batch processing of multiple patient images (Figure S2b).
- **Settings:** The settings button provides options for standardizing images to standard coordinates and applying N4 bias field correction, as discussed in the main manuscript.

Whenever a file is loaded into the toolbox, relevant information about the NIFTI/DICOM file is displayed at the bottom of the screen. This ensures that users have access to essential metadata throughout the segmentation process.

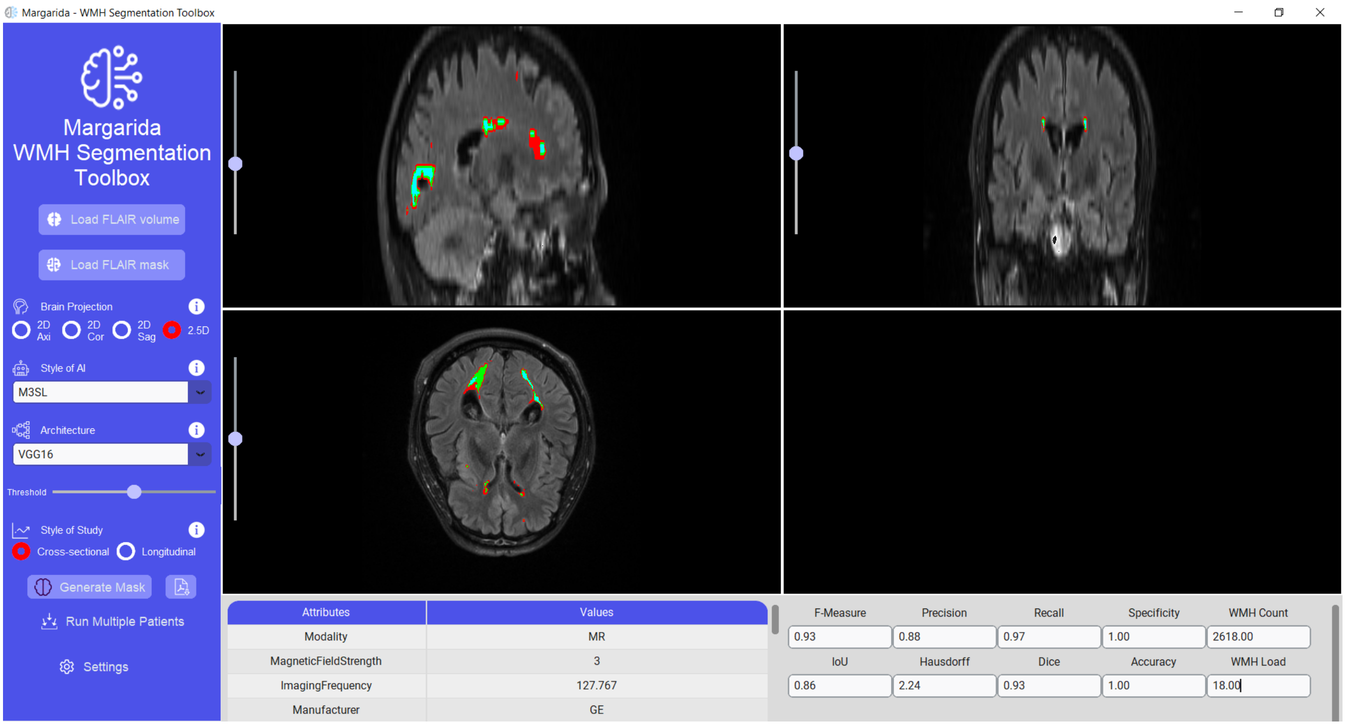

Figure S1. Main screen of the Margarida WMH Segmentation Toolbox. The right panel displays the FLAIR image volume in multiple orientations (axial, coronal, sagittal). The left panel contains the menu options for loading images, selecting segmentation settings, and running the model.

Implementation and Accessibility

The Margarida WMH Segmentation Toolbox is fully implemented in Python and is accessible through the official GitHub repository. The source code, along with pre-trained models, is publicly available to

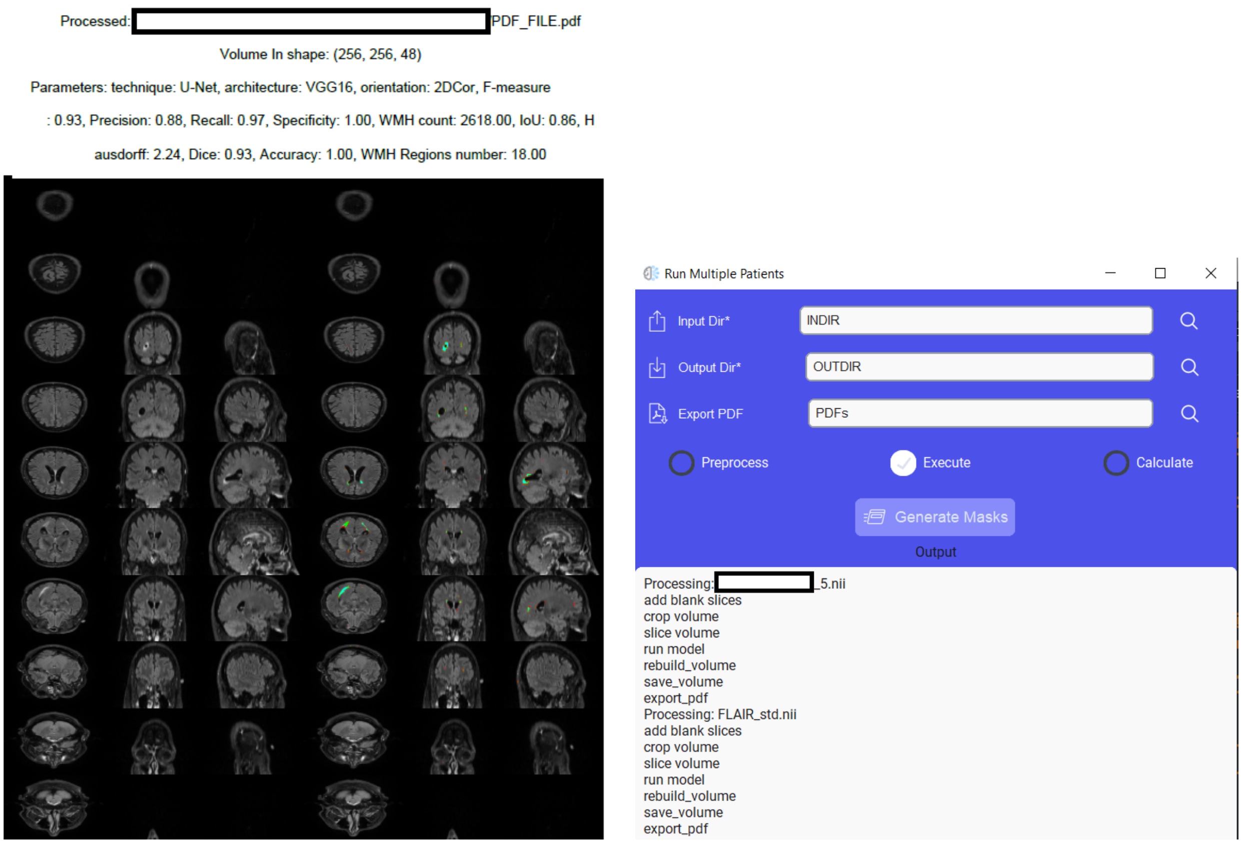

(a) **(b)**

Figure S2. (a) The PDF report generated by the Margarida WMH Segmentation Toolbox. (b) The interface for running the segmentation model on multiple patients simultaneously.

ensure reproducibility and ease of use. The toolbox has been tested across multiple operating systems, including Windows, macOS, and Linux, to ensure broad compatibility.

Users can run the software either through its graphical user interface or via command line (Linux and macOS). Additionally, Docker and Singularity images are provided for streamlined deployment in different computing environments. For detailed guidance on installation and usage, a video tutorial is also available on the GitHub page.

This toolbox represents a significant step forward in automating the segmentation of white matter hyperintensities, offering a user-friendly interface and the flexibility to adapt to various research needs.

FLAIR Image Evaluation Dataset Availability

A subset of FLAIR images from the Calgary Normative Study (CNS) McCreary et al. (2020) is available for use with the Margarida WMH Segmentation Toolbox. This evaluation dataset is specifically prepared for testing and validating segmentation algorithms using the provided software.

The dataset includes a total of 140 images, with 20 images selected per decade from individuals aged 20 to 85 years old. Each decade is balanced for sex (10 male and 10 female individuals). The images were selected following a normal distribution within each age group to provide a representative sample of the population.

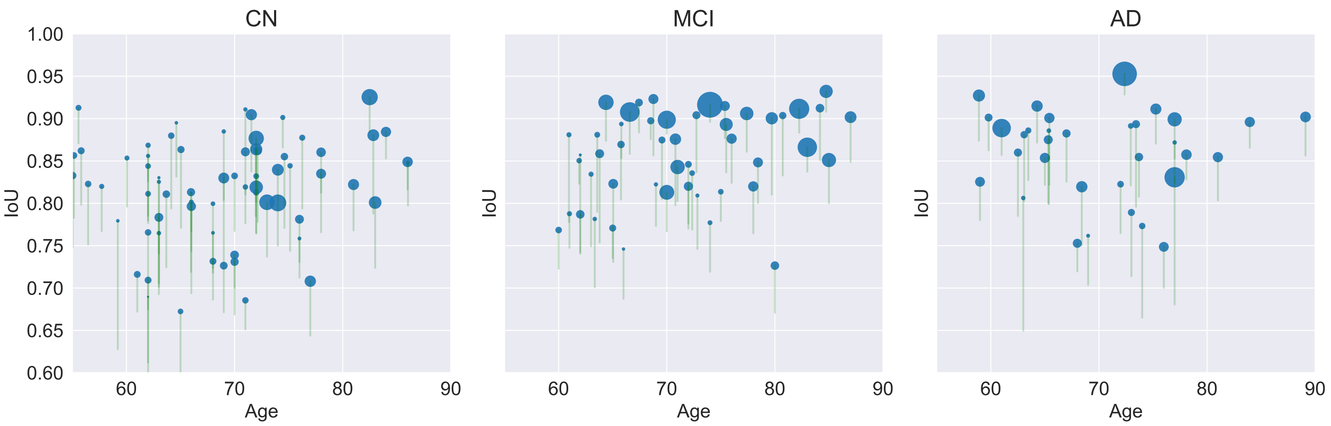

Figure S3. Evaluation of IoU score for the U-Net model across 260 individual subjects over age grouped by clinical stage: (a) CN = cognitively normal (148 individuals), (b) MCI = mild cognitive impairment (55 individuals), and (c) AD = Alzheimer's disease (37 individuals). Size of the filled circle in the plots reflects rank of the normalized WMH volume (*i.e.*, expressed as a percentage of the inter-cranial volume). Green vertical lines represent an increase in IoU values in the same individual, from baseline (bottom of the vertical line) to M3SL variant (top of the vertical line). Conversely, red vertical lines indicate a decrease in IoU for the M3SL compared to baseline variant. For all individuals ($260/260$ [100%]) IoU increased from baseline to M3SL model. This plot confirms the improved performance for M3SL compared to baseline model. Plotted are data from the local datasets described in Table 1.

Alongside the FLAIR images, corresponding manually annotated masks and demographic information (age and sex) are included for each subject. This dataset serves as a valuable resource for researchers and developers to evaluate the performance of their segmentation models.

These images and associated data will be available upon request (refer to Github). This Evaluation Dataset is derived from the Calgary Normative Study, as referenced in McCreary et al. (2020).

2 SUPPLEMENTARY METHODOLOGY

Supplementary Tables are provided to support interpretation of the Figures in the main paper (specifically Figures 2-5 in the main paper).

Supplementary Figures S3 and S4 extend the summary analysis of Figure 6.

Where available, data are report by clinical stage (cognitively normal = CN, mild cognitive impairment = MCI and Alzheimer's disease = AD). Table 1 (see main paper) describes the CN, MCI and AD cohorts within the three local datasets ($n = 260$),McCreary et al. (2020); Peca et al. (2013); Subotic et al. (2021) as well as over the annotated public datasets ($n = 60$).Kuijf et al. (2019)

The pooled group ($n = 320$) in each supplementary table includes both the local datasets and the annotated public dataset.

Results were calculated over voxels containing brain tissue. Normalized WMH (nMWH) reports the fraction of the intracranial (or brain) volume.

Reported are mean $\pm$ standard deviation calculated across subjects.

3 SUPPLEMENTARY RESULTS

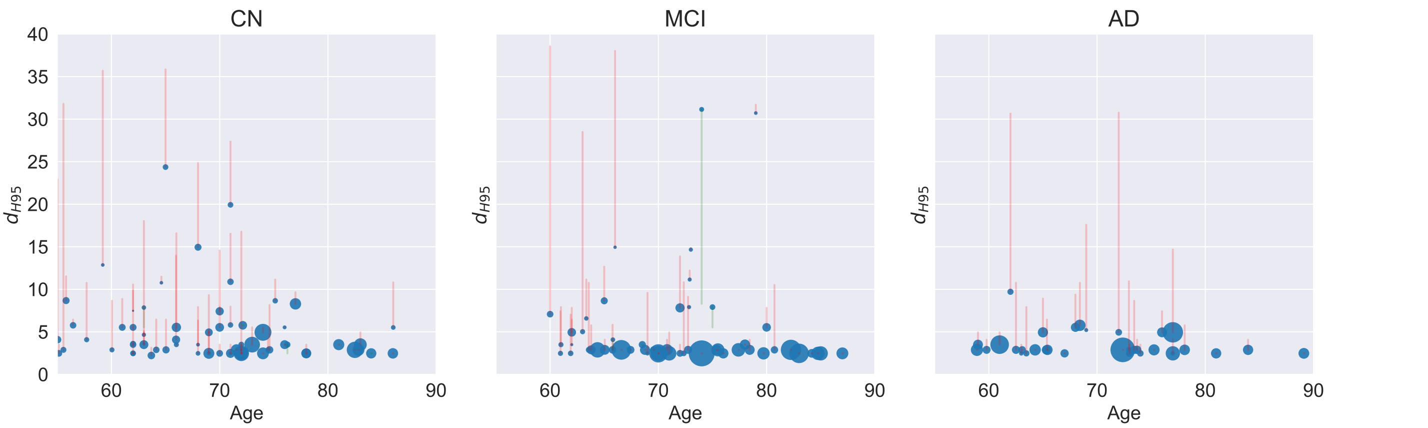

Figure S4. Evaluation of d_{H95} distance for the U-Net model across 260 individual subjects over age grouped by clinical stage: (a) CN = cognitively normal (148 individuals), (b) MCI = mild cognitive impairment (55 individuals), and (c) AD = Alzheimer's disease (37 individuals). Size of the filled circle in the plots reflects rank of the normalized WMH volume (*i.e.*, expressed as a percentage of the inter-cranial volume). Green vertical lines represent an increase in d_{H95} values in the same individual, from baseline (bottom of the vertical line) to M3SL variant (top of the vertical line). Conversely, red vertical lines indicate a decrease in d_{H95} for the M3SL compared to baseline variant. Only $7/260$ (0.027%) of these lines were green. The decrease in d_{H95} (red lines) is desirable since shows an improvement in segmentation. This plot demonstrates better performance for M3SL compared to baseline model. Plotted are data from the local datasets described in Table 1.

Table S1. Normalized WMH volume (nWMH) (mean ± standard deviation) by clinical stage for the ground truth and results obtained by each U-Net model (Multi-Stage Semi-Supervised Learning (M3SL), baseline, transfer learning (TL)). Also shown are results of annotated public dataset and pooled results.

Level	Ground Truth (%)	M3SL (%)	Baseline (%)	TL (%)
CN	0.35 ± 0.37	0.32 ± 0.34	0.31 ± 0.33	0.31 ± 0.33
MCI	0.59 ± 0.74	0.57 ± 0.72	0.56 ± 0.71	0.55 ± 0.71
AD	0.58 ± 0.70	0.56 ± 0.68	0.54 ± 0.65	0.53 ± 0.64
Annotated Public	1.16 ± 1.13	1.04 ± 1.01	0.77 ± 0.85	0.78 ± 0.86
Pooled	0.67 ± 0.84	0.61 ± 0.77	0.53 ± 0.67	0.53 ± 0.67

.

Table S2. Whole brain average false negative (FNF), false positive (FPF) and true positive (TPF) fractions (mean ± standard deviation) by clinical stage for each U-Net model. Also shown are results of annotated public dataset and pooled results. Note: The dataset is imbalanced and in all cases the true negative fraction (TNF) exceeds 0.98

	M3SL			Baseline		
Level	FNF (%)	FPF (%)	TPF (%)	FNF (%)	FPF (%)	TPF (%)
CN	0.05 ± 0.05	0.02 ± 0.02	0.30 ± 0.33	0.06 ± 0.07	0.03 ± 0.03	0.28 ± 0.31
MCI	0.05 ± 0.05	0.03 ± 0.04	0.54 ± 0.70	0.07 ± 0.07	0.04 ± 0.04	0.52 ± 0.68
AD	0.05 ± 0.06	0.02 ± 0.02	0.53 ± 0.66	0.08 ± 0.12	0.04 ± 0.03	0.50 ± 0.63
Annotated Public	0.18 ± 0.19	0.06 ± 0.11	0.97 ± 0.98	0.42 ± 0.52	0.06 ± 0.10	0.71 ± 0.81
Pooled	0.08 ± 0.12	0.03 ± 0.07	0.58 ± 0.74	0.16 ± 0.32	0.04 ± 0.06	0.49 ± 0.64

	TL		
Level	FNF (%)	FPF (%)	TPF (%)
CN	0.07 ± 0.07	0.03 ± 0.03	0.28 ± 0.31
MCI	0.07 ± 0.08	0.04 ± 0.04	0.51 ± 0.67
AD	0.08 ± 0.13	0.04 ± 0.03	0.50 ± 0.62
Public	0.40 ± 0.52	0.06 ± 0.10	0.72 ± 0.82
Pooled	0.16 ± 0.31	0.04 ± 0.06	0.49 ± 0.64

Table S3. Average F-Measure (mean ± standard deviation) by clinical stage for each U-Net model and orientation. Also shown are results of annotated public dataset and pooled results. 2.5D represents the pooled average of the axial, coronal and sagittal predictions.Duarte et al. (2023)

	M3SL				Baseline			
Level	2D Axial	2D Coronal	2D Sagittal	2.5D	2D Axial	2D Coronal	2D Sagittal	2.5D
CN	0.82 ± 0.06	0.87 ± 0.04	0.85 ± 0.05	0.89 ± 0.04	0.74 ± 0.06	0.84 ± 0.04	0.80 ± 0.05	0.85 ± 0.05
MCI	0.84 ± 0.07	0.89 ± 0.05	0.87 ± 0.07	0.90 ± 0.07	0.77 ± 0.07	0.86 ± 0.06	0.83 ± 0.07	0.86 ± 0.07
AD	0.85 ± 0.05	0.89 ± 0.05	0.87 ± 0.06	0.91 ± 0.05	0.78 ± 0.05	0.86 ± 0.07	0.83 ± 0.07	0.87 ± 0.06
Public	0.82 ± 0.07	0.82 ± 0.07	0.80 ± 0.09	0.84 ± 0.07	0.70 ± 0.10	0.72 ± 0.13	0.70 ± 0.14	0.72 ± 0.14
Pooled	0.83 ± 0.06	0.87 ± 0.06	0.84 ± 0.07	0.88 ± 0.06	0.74 ± 0.08	0.81 ± 0.10	0.79 ± 0.10	0.82 ± 0.11

	TL			
Level	2D Axial	2D Coronal	2D Sagital	2.5D
CN	0.74 ± 0.06	0.83 ± 0.05	0.80 ± 0.06	0.84 ± 0.04
MCI	0.76 ± 0.07	0.85 ± 0.05	0.82 ± 0.07	0.86 ± 0.06
AD	0.76 ± 0.06	0.84 ± 0.08	0.83 ± 0.06	0.86 ± 0.07
Annotated Public	0.71 ± 0.10	0.71 ± 0.15	0.69 ± 0.14	0.72 ± 0.14
Pooled	0.74 ± 0.08	0.81 ± 0.11	0.78 ± 0.10	0.82 ± 0.10

.

Table S4. Average IoU (mean ± standard deviation) by clinical stage for each U-Net model and orientation. Also shown are results of annotated public dataset and pooled results. 2.5D represents the pooled average of the axial, coronal and sagittal predictions.Duarte et al. (2023)

	M3SL				Baseline			
Level	2D Axial	2D Coronal	2D Sagital	2.5D				
CN	0.72 ± 0.08	0.79 ± 0.07	0.76 ± 0.08	0.82 ± 0.07	0.61 ± 0.10	0.74 ± 0.07	0.69 ± 0.09	0.75 ± 0.07
MCI	0.75 ± 0.11	0.81 ± 0.09	0.78 ± 0.11	0.83 ± 0.11	0.64 ± 0.12	0.77 ± 0.09	0.74 ± 0.12	0.78 ± 0.11
AD	0.77 ± 0.07	0.82 ± 0.08	0.80 ± 0.08	0.85 ± 0.07	0.66 ± 0.08	0.76 ± 0.12	0.73 ± 0.13	0.78 ± 0.12
Annotated Public	0.73 ± 0.11	0.72 ± 0.12	0.70 ± 0.12	0.75 ± 0.11	0.55 ± 0.16	0.56 ± 0.21	0.55 ± 0.20	0.58 ± 0.21
Pooled	0.74 ± 0.10	0.78 ± 0.10	0.75 ± 0.11	0.81 ± 0.10	0.61 ± 0.12	0.70 ± 0.16	0.67 ± 0.16	0.72 ± 0.16

	TL			
Level	2D Axial	2D Coronal	2D Sagittal	2.5D
CN	0.61 ± 0.10	0.73 ± 0.07	0.69 ± 0.09	0.74 ± 0.07
MCI	0.63 ± 0.12	0.76 ± 0.09	0.72 ± 0.12	0.77 ± 0.11
AD	0.65 ± 0.09	0.75 ± 0.12	0.72 ± 0.13	0.77 ± 0.11
Annotated Public	0.56 ± 0.16	0.57 ± 0.21	0.55 ± 0.20	0.59 ± 0.20
Pooled	0.61 ± 0.12	0.70 ± 0.15	0.66 ± 0.15	0.71 ± 0.15

Table S5. Average Hausdorff 95% distance (d_{H95}, mean ± standard deviation) by clinical stage for each U-Net model and orientation. Also shown are results of annotated public dataset and pooled results. 2.5D represents the pooled average of the axial, coronal and sagittal predictions.Duarte et al. (2023)

Level	M3SL (mm)	Baseline (mm)	TL (mm)
CN	6.75 ± 15.81	9.19 ± 9.74	7.90 ± 6.93
MCI	5.21 ± 5.95	7.91 ± 8.49	7.42 ± 8.18
AD	3.48 ± 1.50	7.28 ± 7.03	7.06 ± 8.46
Annotated Public	14.55 ± 16.60	25.72 ± 18.97	23.17 ± 18.29
Pooled	8.03 ± 13.50	13.11 ± 14.62	11.89 ± 13.54

Table S6. Average F-Measure (mean ± standard deviation) by clinical stage for each U-Net model and scanner type (see description in Table 1). Also shown are results of annotated public dataset and pooled results.

	M3SL				
Level	A	B	C	D	E
CN	0.91 ± 0.03	0.85 ± 0.03	–	–	–
MCI	0.91 ± 0.06	0.85 ± 0.08	–	–	–
AD	0.91 ± 0.06	0.89 ± 0.03	–	–	–
Annotated Public	–	–	0.87 ± 0.06	0.82 ± 0.07	0.82 ± 0.07
Pooled	0.91 ± 0.05	0.86 ± 0.05	0.87 ± 0.06	0.82 ± 0.07	0.82 ± 0.07

	Baseline				
Level	A	B	C	D	E
CN	0.86 ± 0.04	0.81 ± 0.04	–	–	–
MCI	0.87 ± 0.06	0.82 ± 0.09	–	–	–
AD	0.87 ± 0.06	0.85 ± 0.03	–	–	–
Annotated Public	–	–	0.84 ± 0.06	0.73 ± 0.08	0.58 ± 0.13
Pooled	0.87 ± 0.05	0.82 ± 0.05	0.84 ± 0.06	0.73 ± 0.08	0.58 ± 0.13

	TL				
Level	A	B	C	D	E
CN	0.85 ± 0.04	0.80 ± 0.04	–	–	–
MCI	0.86 ± 0.06	0.82 ± 0.07	–	–	–
AD	0.86 ± 0.07	0.84 ± 0.03	–	–	–
Annotated Public	–	–	0.84 ± 0.07	0.74 ± 0.08	0.59 ± 0.13
Pooled	0.86 ± 0.05	0.81 ± 0.05	0.84 ± 0.07	0.74 ± 0.08	0.59 ± 0.13

Table S7. Average IoU (mean ± standard deviation) by clinical stage for each U-Net model and scanner type (see description in Table 1). Also shown are results of annotated public dataset and pooled results.

M3SL

Level	A	B	C	D	E
CN	0.84 ± 0.04	0.74 ± 0.07	–	–	–
MCI	0.84 ± 0.10	0.76 ± 0.14	–	–	–
AD	0.86 ± 0.07	0.81 ± 0.06	–	–	–
Annotated Public	–	–	0.79 ± 0.09	0.74 ± 0.13	0.71 ± 0.11
Pooled	0.85 ± 0.07	0.76 ± 0.09	0.79 ± 0.09	0.74 ± 0.13	0.71 ± 0.11

Baseline

	A	B	C	D	E
CN	0.77 ± 0.06	0.68 ± 0.07	–	–	–
MCI	0.79 ± 0.10	0.72 ± 0.15	–	–	–
AD	0.79 ± 0.12	0.76 ± 0.08	–	–	–
Annotated Public	–	–	0.74 ± 0.10	0.61 ± 0.13	0.38 ± 0.19
Pooled	0.78 ± 0.09	0.70 ± 0.09	0.74 ± 0.10	0.61 ± 0.13	0.38 ± 0.19

TL

	A	B	C	D	E
CN	0.77 ± 0.05	0.66 ± 0.08	–	–	–
MCI	0.78 ± 0.11	0.71 ± 0.13	–	–	–
AD	0.77 ± 0.12	0.74 ± 0.08	–	–	–
Annotated Public	–	–	0.74 ± 0.09	0.62 ± 0.13	0.39 ± 0.19
Pooled	0.77 ± 0.09	0.69 ± 0.10	0.74 ± 0.09	0.62 ± 0.13	0.39 ± 0.19

.

Table S8. Average Hausdorff 95% distance (d_{H95}, mean ± standard deviation) by clinical stage for each U-Net model and scanner type (see description in Table 1). Also shown are results of annotated public dataset and pooled results.

M3SL

	A	B	C	D	E
CN	6.33 ± 17.70	8.17 ± 6.33	–	–	–
MCI	4.62 ± 5.01	8.94 ± 9.89	–	–	–
AD	3.26 ± 1.49	4.77 ± 0.76	–	–	–
Annotated Public	–	–	9.65 ± 8.52	17.36 ± 19.74	16.65 ± 18.83
Pooled	5.03 ± 11.89	7.76 ± 6.80	9.65 ± 8.52	17.36 ± 19.74	16.65 ± 18.83

Baseline

	A	B	C	D	E
CN	8.15 ± 9.68	12.69 ± 9.41	–	–	–
MCI	7.33 ± 8.26	11.61 ± 9.71	–	–	–
AD	6.97 ± 7.47	9.06 ± 3.58	–	–	–
Annotated Public	–	–	14.31 ± 12.02	22.66 ± 13.34	40.97 ± 20.46
Pooled	7.60 ± 8.67	11.77 ± 8.60	14.31 ± 12.02	22.66 ± 13.34	40.97 ± 20.46

TL

	A	B	C	D	E
CN	6.29 ± 4.91	13.30 ± 9.75	–	–	–
MCI	6.67 ± 7.91	12.25 ± 8.92	–	–	–
AD	6.83 ± 9.11	8.37 ± 2.50	–	–	–
Annotated Public	–	–	12.16 ± 9.42	21.02 ± 13.72	36.29 ± 22.32
Pooled	6.55 ± 7.08	12.16 ± 8.65	12.16 ± 9.42	21.02 ± 13.72	36.29 ± 22.32

REFERENCES

Duarte, K. T., Gobbi, D. G., Sidhu, A. S., McCreary, C. R., Saad, F., Camicioli, R., et al. (2023). Segmenting white matter hyperintensities in brain magnetic resonance images using convolution neural networks. *Pattern Recognition Letters* 175, 90–94. doi:10.1016/j.patrec.2023.07.014

Kuijf, H. J., Casamitjana, A., Collins, D. L., Dadar, M., and et al., A. G. (2019). Standardized assessment of automatic segmentation of white matter hyperintensities and results of the WMH segmentation challenge. *IEEE Transactions on Medical Imaging* 38, 2556–2568. doi:10.1109/tmi.2019.2905770

McCreary, C. R., Salluzzi, M., Andersen, L. B., Gobbi, D., Lauzon, L., Saad, F., et al. (2020). Calgary Normative Study: Design of a prospective longitudinal study to characterise potential quantitative MR biomarkers of neurodegeneration over the adult lifespan. *BMJ Open* 10. doi:10.1136/bmjopen-2020-038120

Peca, S., McCreary, C. R., Donaldson, E., Kumarpillai, G., Shobha, N., and et al (2013). Neurovascular decoupling is associated with severity of cerebral amyloid angiopathy. *Neurology* 81, 1659–1665. doi:10.1212/01.wnl.0000435291.49598.54

Subotic, A., McCreary, C., Saad, F., Nguyen, A., Alvarez-Veronesi, A., and et al (2021). Cortical thickness and its association with clinical cognitive and neuroimaging markers in cerebral amyloid angiopathy. *Journal of Alzheimer's Disease* 81, 1–9. doi:10.3233/JAD-210138
